# Transcriptional Profiling of Nitrogen Fixation and the Role of NifA in the Diazotrophic Endophyte *Azoarcus* sp. Strain BH72

**DOI:** 10.1371/journal.pone.0086527

**Published:** 2014-02-06

**Authors:** Abhijit Sarkar, Barbara Reinhold-Hurek

**Affiliations:** University of Bremen, Faculty of Biology, Department of Microbe-Plant Interactions, Bremen, Germany; Agriculture and Agri-Food Canada, Canada

## Abstract

**Background:**

The model endophyte *Azoarcus* sp. strain BH72 is known to contribute fixed nitrogen to its host Kallar grass and also expresses nitrogenase genes endophytically in rice seedlings. Availability of nitrogen is a signal regulating the transcription of nitrogenase genes. Therefore, we analysed global transcription in response to differences in the nitrogen source.

**Methodology/Principal Findings:**

A DNA microarray, comprising 70-mer oligonucleotides representing 3989 open reading frames of the genome of strain BH72, was used for transcriptome studies. Transcription profiles of cells grown microaerobically on N_2_ versus ammonium were compared. Expression of 7.2% of the genes was significantly up-regulated, and 5.8% down-regulated upon N_2_ fixation, respectively. A parallel genome-wide prediction of σ^54^-type promoter elements mapped to the upstream region of 38 sequences of which 36 were modulated under the N_2_ response. In addition to modulation of genes related to N_2_ fixation, the expressions of gene clusters that might be related to plant-microbe interaction and of several transcription factors were significantly enhanced. While comparing under N_2_-fixation conditions the transcriptome of wild type with a *nifLA^−^* insertion mutant, NifA being the essential transcriptional activator for *nif* genes, 24.5% of the genome was found to be affected in expression. A genome-wide prediction of 29 NifA binding sequences matched to 25 of the target genes whose expression was differential during microarray analysis, some of which were putatively negatively regulated by NifA. For selected genes, differential expression was corroborated by real time RT-PCR studies.

**Conclusion/Significance:**

Our data suggest that life under conditions of nitrogen fixation is an important part of the lifestyle of strain BH72 in roots, as a wide range of genes far beyond the *nif* regulon is modulated. Moreover, the NifA regulon in strain BH72 appears to encompass a wider range of cellular functions beyond the regulation of *nif* genes.

## Introduction

Biological nitrogen fixation, involving the enzymatic conversion of atmospheric nitrogen to ammonia by nitrogenase, is an important process to maintain soil fertility and life on earth, counterbalancing loss of nitrogen *e.g.* by denitrification. It is catalyzed by the two-component nitrogenase complex restricted to prokaryotes, the reaction being oxygen-sensitive demanding high amounts of energy and reductants. Nitrogen-fixing microorganisms encompass a broad habitat range from free living forms in soils and water to endophytic association with grasses or in root-nodule symbioses with legumes. Consequently, they have evolved sophisticated regulatory networks that respond to multiple environmental cues [1]. Regulation of *nif* gene expression has been most extensively studied in diazotrophic *Proteobacteria* [2]. Albeit the genes necessary for nitrogen fixation in many diazotrophs have common structures and functions, the mechanisms by which cellular nitrogen levels are sensed and nitrogen signals are transmitted can vary considerably among different nitrogen-fixing bacteria [3]. Oxygen and fixed nitrogen, such as ammonium, are the important environmental signals that regulate nitrogen fixation.

*Azoarcus* sp. strain BH72 is a diazotrophic model endophyte of grasses [4], belonging to *Betaproteobacteria* [5]. N_2_ fixation and *nifHDK* transcription occur only under microaerobic and nitrogen-limiting conditions in this strictly respiratory bacterium [6], [7]. The structural genes of nitrogenase *nifHDK* were even found to be expressed and translated in the aerenchyma of rice seedlings [6], [8], and fixed nitrogen can be contributed in an unculturable state to the host plant Kallar grass [9].

*Azoarcus* sp. strain BH72 is apparently highly adapted to environments poor in available nitrogen sources, which correlates with its role as an N_2_-fixing endophyte. (i) A low-affinity glutamate dehydrogenase (GDH) for ammonium assimilation is lacking, a feature highly unusual in free-living bacteria; only the high-affinity ATP- consuming assimilation system (GS[2x]-GOGAT) is present [10]. (ii) Four genes encoding putative high-affinity ammonia transporters exist (*amtB/Y/D/E*), one of them with an additional regulatory domain [11]. (iii) Single copies of structural genes for the molybdenum-dependent nitrogenase complex and all genes required for cofactor synthesis and maturation of the nitrogenase are present in strain BH72 [10]. (iv) A battery of electron transporters which might contribute to nitrogenase activity is encoded in the genome, including two flavodoxins (*nifF1, nifF2*), 12 genes for ferredoxin-like proteins, and two gene clusters for putative electron transport systems to ferredoxins (*rnf1, rnf2*) [11]. Thus, nitrogen fixation appears to be an important part of the lifestyle of this bacterium, however genome-wide expression profiles are not known under these conditions.

In *Azoarcus* sp. strain BH72 and other nitrogen-fixing symbiotic bacteria, RpoN and NifA are master regulators of nitrogen fixation genes. The alternative sigma factor 54 (RpoN) is employed by many bacteria to transcribe genes involved in a wide variety of cellular functions such as nitrogen utilization [12], virulence, stress responses [13] and flagellum biosynthesis [14]. RpoN binds to -24/−12-type promoters with consensus sequence TGGCACG-N4-TTGC [15], [16]. However, for initiation of transcription it requires additionally an Enhancer Binding Protein (EBP), such as the transcriptional regulator NifA [1], [17]. Also in *Azoarcus* sp. strain BH72, NifA plays a central role as transcriptional activator for nitrogenase gene (*nifHDK*) expression [18]. As otherwise only reported for *Gammaproteobacteria* [19], in strain BH72 an additional regulatory protein NifL is involved as an anti-activator of NifA that regulates its activity in response to oxygen and combined nitrogen [18]. However, the entire regulon of the NifLA system has not yet been determined.

In order to characterize the nitrogen response of *Azoarcus* sp. strain BH72, we used a genome-wide microarray [20] to analyze the transcriptome. Besides for typically expected *nif* genes, modulation of expression was observed under nitrogen fixation versus N-replete conditions for several non-*nif* genes as well as genes encoding for hypothetical protein(s). In a parallel genome wide *in silico* approach, several corresponding RpoN and NifA binding sites were predicted. Transcriptome profiling in a *nifLA* insertional mutant revealed a relatively large regulon indirectly or directly affected by NifLA.

## Results and Discussion

### General Nitrogen Response of Strain *Azoarcus* sp. Strain BH72

**Figure 1:**
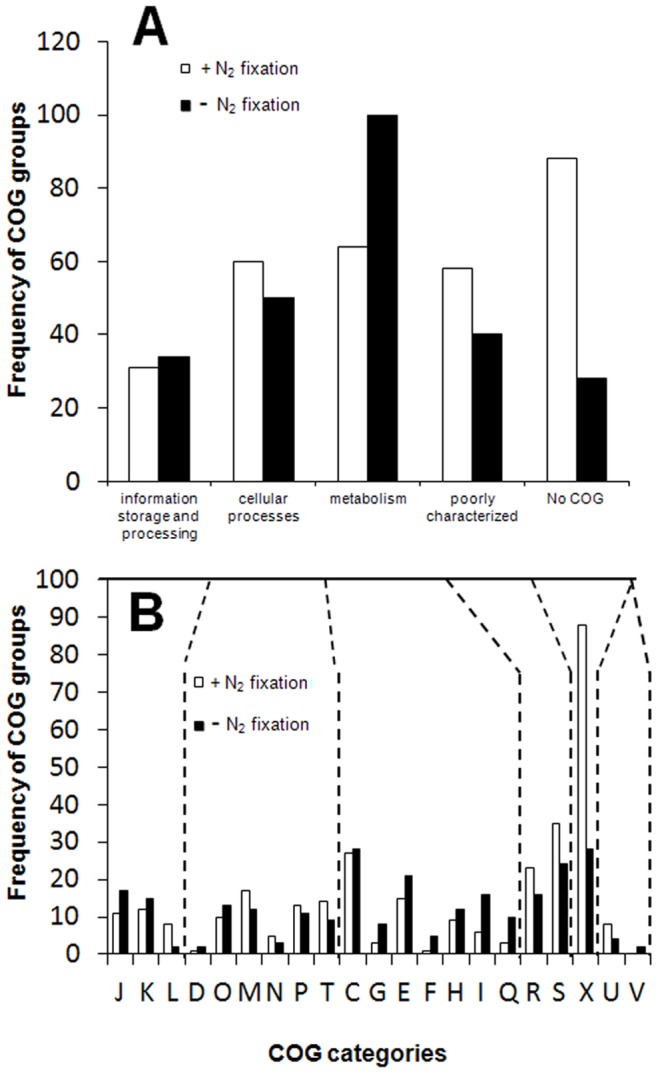
Distribution of differentially regulated genes of *Azoarcus* sp. strain BH72 according to COG categories. Genes up regulated under N_2_ fixation shown by white bars (N_2_)and those down regulated under N_2_ fixation shown by black bars (NH_3_). (A) Broad COG categories; (B) subcategories within a broad category. C: Energy production and conversion, D: Cell cycle control, mitosis and meiosis, E: Amino acid transport and metabolism, F: Nucleotide transport and metabolism, G: Carbohydrate transport and metabolism, H: Coenzyme transport and metabolism, I: Lipid transport and metabolism, J:Translation, K: Transcription, L: Replication, recombination and repair, M: Cell wall/membrane biogenesis, N: Cell motility, O: Posttranslational modification, protein turnover, chaperones, P: Inorganic ion transport and metabolism, Q: Secondary metabolites biosynthesis, transport and catabolism, R: General function prediction only, S: Function unknown, T: Signal transduction mechanisms, U: Intracellular trafficking and secretion, X: not in COG.

**Figure 2:**
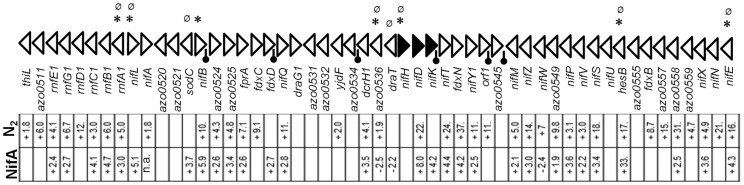
Schematic representation of the core *nif* gene cluster (*azo0510*- *azo0562*) and its expression in *Azoarcus* sp. strain BH72. The *nif* structural genes *nifH, nifD* and *nifK* are represented by solid triangles. Black bars with round caps when denoted in the intergenic region represent transcription termination loops. The symbols ∗ or Ø on top of a triangle denotes the occurrence of predicted RpoN-binding site or NifA binding site upstream of that particular target gene, respectively. Upper row (N_2_) represents fold change of expression of genes of the *nif* cluster from triplicate microarray experiments either up-regulated (+) or down-regulated (−) under N_2_ fixation. Lower row (NifA) represents fold change of expression of the genes of the *nif* cluster either positively (+) or negatively (−) regulated by NifA. Empty rectangles below a gene represent no significant modulation of expression of that gene of interest under one or both of the microarray experiments. N. a., not applicable in case of *nifA* mutant.

In the order to analyze the nitrogen response of *Azoarcus* sp. strain BH72, wild type cells were grown under microaerobic conditions either on N_2_ or on combined nitrogen (ammonium chloride). The global gene expression profile was compared by oligonucleotide-based transcriptome microarray studies. A total of 524 (13.1%) genes exhibited more than a 1.8 fold change in expression under nitrogen fixation in comparison to N-replete conditions (*P<*0.05). This cut-off was used as it allowed better coverage of cotranscribed genes, some of which were only moderately regulated. Among these genes, the expression of 290 (7.2%) genes was enhanced and the expression of 234 (5.8%) genes was repressed (see Table S1, Figure 1) under N_2_ fixation. The genome of strain BH72 harbors 55 genes (*azo0510 to azo0564*) located in a *nif* cluster encoding for proteins directly or indirectly involved in nitrogen fixation according to their annotation [10], accounting only about 1.3% of the protein encoding genes (Figure 2). Therefore, the N_2_ response in strain BH72 elicited the expression of a 10-fold higher number of genes even outside the *nif* cluster. Selected results obtained by the microarray approach were further examined by real-time RT-PCR. Similarly, 549 out of 4146 predicted genes had enhanced expression under nitrogen fixation in *Pseudomonas stutzeri* A1501, accounting about 13% [21]. For *Azotobacter vinelandii*, transcript levels for even 30% of the genes changed more than two-fold during diazotrophic growth compared to the N-replete control [22].

**Figure 3:**
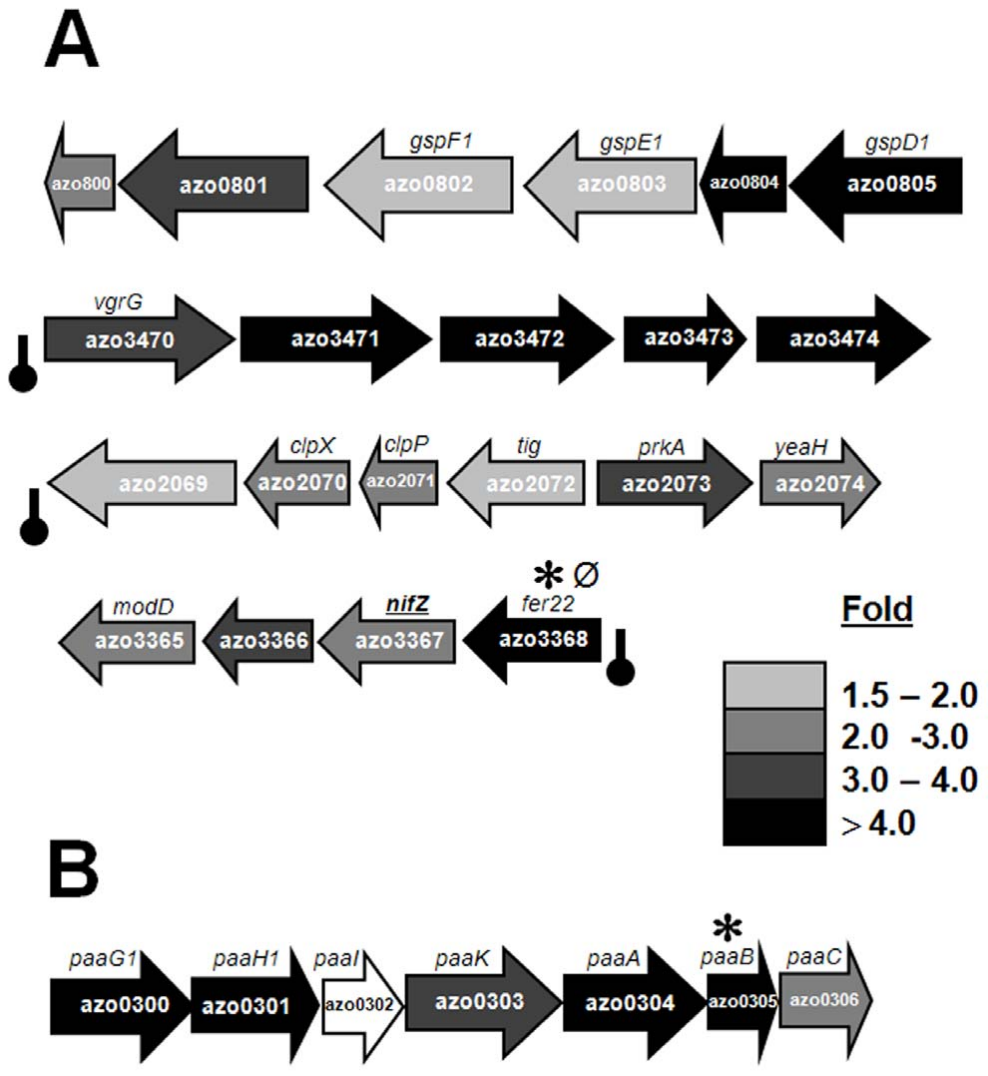
Selected gene clusters up-regulated or down-regulated under N_2_ fixation in comparison to growth on ammonia. (A) Clusters up-regulated, with following functions: gene cluster encoding for components of general secretion pathway (type II); cluster encoding for several hypothetical proteins, the first gene in the cluster being *vgrG* (component of type VI secretion); cluster of genes encoding for proteases; cluster harboring genes involved in *nif* maturation, *nifZ*, ferredoxin like mobile electron carrier *fer22*, and component of Molybdenum transport *modD;* closely linked genes within the *nif* cluster encoding for FeMoco cofactor biosynthesis protein, hypothetical protein, sigma E factor regulatory protein and probable ferrodoxin. (B) Cluster down-regulated under N_2_ fixation; genes encoding for proteins/enzymes involved in phenylacetic acid degradation pathway (*paa*). Fold change represented by shades of grey give the average fold of induction from 3 independent microarray experiments in each case. Black bar with round cap, symbols ∗ or Ø, represent intergenic transcription termination loop, putative RpoN-binding sites or putative NifA-binding sites, respectively, upstream of the target gene of interest as described previously in the legend of Figure 2.

Genes modulated under N_2_ fixation were evenly distributed across most general COG functional categories (**C**lusters of **O**rthologus **G**roup(s)), however the group belonging to “not in COG” was most frequently up-regulated (Figure 1, Table S1). Accordingly, 28% of the up-regulated genes and 12% of down-regulated genes under N_2_ fixation belonged to this group. Even several gene clusters with unknown functions exhibited enhanced expression under N_2_ fixation, such as cluster *azo1297-azo1310* or cluster *azo3470-azo3474* (Figure 3, Table S1). Interestingly, recently it has been shown that some of them encode components of a type VI secretion system (see below), [23].

The group of metabolism-related genes represented an also frequently modulated COG category. However, the majority of the members were down-regulated under N_2_ fixation, particularly those belonging to carbohydrate, coenzyme, lipid and secondary metabolite metabolism subgroups, respectively (Figure 1). COG U represented by proteins involved in secretion was also strongly overrepresented under N_2_ fixation. Interestingly, genes from the subcategory L representing replication, recombination and repair proteins were mainly up-regulated under N_2_ fixation, while members representing transcription (K) as well as translation, ribosomal structure and biogenesis subfamilies (J) were mainly found to be down-regulated (Figure 1).

### Differential Expression of Genes Related to Nitrogen Fixation

Several gene clusters were found to be significantly up-regulated under N_2_ fixation, indicating functional relationships. They can be divided into two broad categories. One of them included genes which are directly or indirectly related to nitrogen fixation represented by gene clusters involved directly in nitrogenase synthesis, maturation and function. As expected, up-regulation of most genes of the *nif* cluster (*azo0512– azo0562*) was detected, with genes encoding proteins involved in nitrogenase maturation, nitrogenase enzyme and electron transporters (Figure 2; Table S1). Although *nifH* up-regulation was not found to be statistically significant due to spot inhomogeneity, it was shown previously that the expression of *nifH*, *fdxN,* and *nifLA* were elevated under N_2_ fixation [11], [24]. Interestingly, specifically the components of *rnf1*-complex encoded within the *nif* cluster and not genes of the *rnf2* complex exhibited enhanced expression under N_2_ fixation. The Rnf1 complex has been reported to couple the energy of ion transport to reduce ferredoxin, and in strain BH72 it appears to play a role in electron transfer to nitrogenase and in regulation of the “switch-off” of nitrogenase in response to ammonia [11]. In addition, *nif* genes outside the *nif* cluster and in distant locations of the chromosome were differentially regulated too; *nifF1* (*azo0014*) and *nifZ* (*azo3367*) along with *fer22* (*azo3368*) (related to nitrogenase maturation) were also induced 2.7, 2.6, 11.5 fold, respectively (Figure 3, Table S1). Likewise, genes encoding for proteins related to efficient N_2_ fixation electron transfer, ATP synthesis and molybdopterin biosynthesis, an important component of FeMo-co, exhibited enhanced expression under nitrogen fixation, respectively (Table S1). Accordingly, genes for molybdenum transporters like *modA1* and *modE* were up-regulated. Also several genes coding for electron transfer flavoproteins like *etf1*, *etfA2*, *etfB2, etfA3*, *etfB3,* flavodoxin *isiB*, probable cytochrome *cc42* and even *nifY2* (nitrogenase maturation protein) were up-regulated. Recently it has been reported that NifB and NifEN protein levels are regulated by protease ClpXP under N_2_ fixation conditions in *A. vinelandii* [25]. This ClpX protein of *A. vinelandii* has highest similarity (76% identical, 84% similar) to the respective ClpX copy (*azo2070*) of *Azoarcus* sp. strain BH72. Concordantly, *clpX* and *clpP* expressions were enhanced by 2 fold in strain BH72 under N_2_ fixation, as well (Figure 3).

### Nitrogen Response of Gene Clusters Not Related to Nitrogen Fixation

Several of the gene clusters differentially regulated in response to nitrogen fixation conditions encoded proteins that were not obviously related to nitrogen metabolism but with completely different functions. This may in part reflect the difference in growth conditions and generation times, although they do not vastly differ (2.10±0.09 on N_2_, 1.66±0.13 on NH_4_^+^) [26].

Transport and secretion: Several secretion clusters were found to be induced under N_2_ fixation in strain BH72. In agreement to the up-regulation of genes in COG group U (Figure 1), a gene cluster encoding for putative components of a type II secretion system (*azo0801– azo0805*) components, was strongly up-regulated under N_2_ fixation (Figure 3; Table S1). For confirmation of differential gene expression in unexpected cases such as this (see also below), an independent method was used. Quantitative RT-PCR analysis validated the induced expression of *azo0805* by even 21.4±5.7 fold under N_2_ response. Proteins transported by the type II secretion pathway have to first translocate across the cytoplasmic membrane via the Sec system and then fold into a translocation-competent conformation in the periplasm. Secreted proteins may include proteases, cellulases, pectinases, phospholipases, lipases, and toxins [27]. This appeared to be a relatively specific induction, as a second type II secretion *gsp* gene cluster (*azo2097- azo2084*) was not affected. Another type of protein secretion system was induced under N_2_ fixation. This cluster was characterized to encode components of type VI secretion system (*azo1299 to azo1307*) [23] (Table S1). Type VI secretion system gene clusters contain from 15 to more than 20 genes, two of which encode Hcp and VgrG, that are nearly universally secreted components of the system [28], [29]. As in several symbiotic or pathogenic interactions with eukaryotes [30], the type VI protein secretion appears to play a role also in *Azoarcus-*rice interactions [23].

Nitrogen and carbon metabolism: As expected, in response to N_2_ fixation the transcription of *glnA* (encoding glutamine synthetase) was enhanced to assimilate the fixed nitrogen, *glnP* and *glnH* (glutamine transporters) were induced, as well (Table S1). In contrast, the transcription of denitrification genes like *napD1* and *napE* (Table S1 *),* as well as a gene for NO_2_^−^ assimilation *nirB* (Table S1), were strongly repressed under N_2_ fixation. Interestingly, expression of *hoxB* encoding for the small subunit of hydrogenase was enhanced by 6.57 fold (Table S1) specifically under N_2_ fixation. It may be tempting to speculate that H_2_ liberated as a by-product under N_2_ fixation by nitrogenase can be further oxidized by an uptake hydrogenase to recycle reducing equivalents.

N_2_ fixation in *Azoarcus* sp. strain BH72 was accompanied by down-regulation of the expression of several of the Embden-Meyerhof pathway (EMP) and tricarboxylic acid cycle (TCA cycle) enzymes. Especially the transcription of the components of PDH complex as well as components of ethanol oxidation pathway producing acetyl-CoA was reduced. Expression of some enzymes of the ß oxidation of the fatty acid pathway generating acetyl-CoA as end product, were also found to be transcriptionally repressed under N_2_ fixation. Perhaps occurrence of high energy charge (ATP/ADP) in N_2_ fixing cells inhibits some of the enzymes and also their expression or this might reflect slower growth.

Translation and transcription: Expression of gene coding for small subunit of ribosomal protein (*azo3394*) as well as translation initiation factors and elongation factors (*azo3419*; *azo3431*) were down-regulated under N_2_ fixation (Table S1), which might reflect the slower growth rate. This is in agreement with the generation time measured for *Azoarcus* sp. strain BH72 under N_2_ fixation (2.10 h) as compared to that in presence of ammonia (1.12 h). The fold of repression (2.9–fold) of one of the ribosomal protein encoding genes, *azo0720* under nitrogen response even corroborated with qRT-PCR approach (−2.5±1.5 fold) under similar condition (Table 1). Consistently, expression of genes encoding DNA directed RNA polymerase subunits, *rpoC*, *rpoB*, and more general sigma factors *rpoH* (sigma32), and *rpoD* (sigma70), as well as several transcription factors like *gacA* and *uidR*, were strongly repressed under N_2_ fixation. In contrast, expression of *algU* (*rpoE*) (sigma 24) and *rpoN2* (sigma 54), but not *rpoN1*, were strongly enhanced under N_2_ fixation (Table S1). Among these two copies in the genome of strain BH72, *rpoN1* (*azo0504*) is located in proximity to the *nif* cluster, and *rpoN2* (*azo1790*) widely distant from the *nif* cluster. A few organisms may have two copies of *rpoN*, such as *Bradyrhizobium japonicum*, *Rhizobium etli*, [31,32], *Ralstonia solanacearum* and *Burkholderia fungorum,* while *Rhodobacter sphaeroides* harbors 4 copies [33]. Generally RpoN1 is involved in the expression of the genes required for nitrogen fixation, whereas RpoN2 is required for the transcription of the class II and class III flagellar genes. The significant induction of *azo1790* by 3.9 -fold (Table S1) was also verified by real time qRT-PCR (17.83±2.94 fold) (Table 1). In *Rhizobium etli*, *rpoN1* expression was negatively autoregulated under aerobic growth and reduced during microaerobiosis and symbiosis while *rpoN2* expression was specifically induced under microaerobiosis and in bacteroids [32]. *R. sphaeroides* harbours 4 copies of *rpoN* gene. *rpoN1* located within *nif* cluster was down-regulated under shift to aerobic conditions while *rpoN2* located with the flagellar genes was transiently up-regulated after shift to aerobiosis [34]. Additionally, a high number of transcription factors belonging to Fis, LysR, TetR, MerR, AraC, and LuxR families respectively were modulated in their expression: 10 were up-regulated and 11 down-regulated; one of them, *azo1584* encoding a hybrid sensor-response regulator, exhibited even a relatively high 6.34 fold enhanced expression on N_2_ (Figure S4). Thus, complex changes in transcriptional regulation appear to occur under conditions of nitrogen fixation. In *Pseudomonas stutzeri*, expression of only 6 transcription factors of the LysR, TetR or AraC family were up-regulated under N_2_ fixation [21]. In *Azotobacter vinelandii*, an Fnr like negative transcriptional regulator of CydAB was up-regulated while IscR (Fe-S assembly Factor) was down-regulated under all conditions of N_2_ fixation with *nifH*, *vnf*, and *anf* [22].

**Table 1:** Differential gene expression of *Azoarcus* sp. BH72 under N_2_ fixation or under NifA regulation detected by real-time PCR and microarray transcriptome studies.

			N_2_ response (induction)		NifA regulon (induction)	
locus tag	gene	gene product	Microarray	qRT-PCR	Microarray	qRT-PCR
azo3471		conserved hypothetical protein	11.1	21.2±9.6	−3.1^a^	−3.2±1.9
azo0954	*isiB*	probable flavodoxin	13.4	60±14.3	3.9	12.6±4.6
azo3368	*fer22*	probable ferredoxin 2Fe-2S protein	11.5	90.7±15.4	4.9	19.9±8.6
azo0804		hypothetical protein	4.1	20.3±2.3	−2.2	−6.2±3.8
azo1790	*rpoN2*	RNA polymerase, sigma 54 subunit, (SigL)	3.9	17.83±2.9	−4.4	−26.3±9.7
azo0805	*gspD1*	general secretion pathway protein D	10.0	21.4±5.7	n/a	n/a
azo1566	*clpB1*	probable ATP-dependent Clp protease.	−4.2	−16.3±6.4	n/a	n a
azo1625	*fabG1*	3-oxoacyl-[acyl-carrier-protein] reductase	−3.6	−3.8±1.4	3.6	52.4±16.6
azo0720	*rpsR*	SSU ribosomal protein S18P	−2.9	−2.5±1.5	−3.1	−4±1.2
azo0585	*tlyA*	conserved hypothetical protein	n/a	n/a	6.1	12.7±3.2
azo1077	*cysM*	cysteine synthase	n/a	n/a	3.3	14.6±5.2
azo3155	*ilvH*	acetolactate synthase. small subunit	n/a	n/a	−5.5	−1.9±0.3
azo3246	*fadD4*	Long-chain-fatty-acid-CoA ligase	n/a	n/a	−3.0	−3.7±1.1

a (–) Represents down-regulated under N_2_ fixation or negatively regulated by NifA.

Secondary metabolism: Flagella and pili are important for microbial colonization of the host, particularly for endophytic colonization of rice roots by *Azoarcus* sp. strain BH72 [35], [36], [37]. Enhancement of expression of *pilH*, *pilY1B* and *fliF* under diazotrophic growth (Table S1) speaks in favor of a common mode of gene regulation. Indeed several of the pilin as well as flagellar genes are known to be under the common control of RpoN along with the *nif* related genes in other bacteria. One of the interesting observations under N_2_ fixation was the strong down-regulation of proteins involved in phenyl acetic acid (PAA) degradation pathway (*paa*-cluster) (Table S1, Figure 3). In *Azospirillum brasilense*, PAA has been reported as an auxin like molecule with anti-microbial activity [38]. It has been reported to play a role in plant growth promotion and protect the producing strain from other competing strains in natural environments. Recently, connections between virulence and gene products of the phenylacetate catabolism have been shown in *Burkholderia cenocepacia* [39]. The accumulation of the early products of PAA catabolism is known to have toxic effects on the host showing disease symptoms. Ring-1,2-epoxide and its phenolic breakdown product 2-hydroxyphenylacetate are obvious candidates for causing such damage [40]. PAA might play a role in rhizosphere competence of *Azoarcus* sp. strain BH72 outside the root, and down-regulation of the PAA degradation pathway under N_2_ fixation might be beneficial for strain BH72 to establish a successful endophytic colonization as a “disarmed plant pathogen” inside rice root without showing signs of disease. Thus, modulation of expression of genes related to plant colonization might link the diazotrophic with the endophytic life style.

### Genome Wide Prediction of −12/−24 Promoters for σ^54^ Factor (RpoN) Binding and of NifA Binding Sites

The RpoN regulon was analyzed in several organisms, such as *E. coli* [41], *Pseudomonas putida* [42] and several species of *Rhizobiaceae* [43] by use of powerful computational methods that took advantage of the high conservation of σ^54^-type promoter sequences throughout diverse bacterial groups. To potentially discriminate between genes directly and indirectly regulated by RpoN and to identify other members of the RpoN regulon undetected by microarray analysis, we carried out an *in silico* search to locate potential RpoN-binding sites in *Azoarcus* sp. strain BH72 genome using the online tool for the prediction of prokaryotic promoter elements and regulons, PePPER: a web based regulon, TF (**T**ranscription **F**actor), and TFBS (**T**ranscription **F**actor **B**inding **S**ite) mining system. The intergenic regions of the complete genome sequence of strain BH72 were scored against position-specific weight matrices (PWM) derived from selected RpoN-binding sites of bacterial species listed in the TFBS tool box of PePPER. With the default setting parameters of the online analysis tool, we were initially able to detect genome wide 173 putative RpoN-binding sites or σ^54^-type promoters upstream of 162 target genes that could potentially direct the transcription of a gene in the correct orientation. The binding sequences of *Azoarcus* sp. strain BH72 were assembled together to generate a genome wide RpoN-binding consensus using Web logo (Figure S1). Restricting the list to those target genes which were also modulated in their expression under N_2_ fixation in microarray experiments, 38 RpoN-binding motifs were predicted to be present upstream of 36 target genes (Table S2). This probably comprised the subset of the whole RpoN regulon involved in controlling functions related to N_2_ fixation accounting to 22% of all genes with predicted RpoN binding motif. Among them, genes *nifB* (*azo0523*) and *narK* (*azo1288*) were with tandem promoter sites as also detected for example for *P. putida* Ca-3 [44]. RpoN binding motifs were predicted upstream of *nifH* and *nifL,* respectively, which have been previously shown experimentally to be modulated under N_2_ fixation [18], [24]. Hence they were additionally included in the list as well, although modulation of *nifH* and *nifL* expression was not detected under current microarray experiments (Table S2). Similarly a candidate gene *pilA*, which is not related to N_2_ fixation but known to possess a RpoN-binding motif [35], was detected by the online analysis tool, speaking in favor of its robustness. As expected, most of the listed genes belonged to N_2_ fixation related functions: structural gene of nitrogenase *nifH* (*azo0538*), maturation process of nitrogenase like *nifB* (*azo0523*), *nifE* (*azo0562*) or electron transport like *rnfA1* (*azo0517*), *nifF1* (*azo0014*), *fer22* (*azo3368*) or transcription factors *rpoN2* (*azo1790*), *azo2932*. Others included those that are involved in nitrogen assimilation, *glnA* (*azo0738*), nitrate transport *narK* (*azo1288*) or even outer membrane efflux proteins as *nodT* (*azo2554*). Outside N_2_ fixation related functions, genes encoding for hypothetical proteins with enhanced expression under N_2_ fixation were also found to be preceded by RpoN-binding motifs. This included 17 different hypothetical protein encoding genes like *azo2488, azo2922, azo2958, azo3080*. 5 out of 17 encoded even secretory proteins (*azo2346*, *azo3866*, *azo3738*, *azo3114,* and *azo3866*). Additionally, ethanol metabolism, with expression of the aldehyde dehydrogenase gene *aldA* (*azo2939*) [45] being repressed under N_2_ fixation, was also found to be linked with an RpoN binding site.

Additionally, binding sites for NifA, the transcriptional activator for *nif* genes, were predicted using the consensus TGT-N_10_-TCA in the intergenic region of the *Azoarcus* sp. strain BH72 (Prodoric). Sequences were searched in both the strands, relative to the context of the gene start spanning up to 500 bases. Sequences generated with a score above -6.5 with their associated target genes exhibiting NifA-regulated differential expression pattern in microarray experiments were chosen for further analysis. In this way, 29 NifA binding sequences were predicted upstream of 25 target genes (Table S3). Tandem NifA binding sequences were found to be present upstream of *rnfA1*, *nifL*, *sodC* and *nifE,* respectively (Table S3). 18 of the listed genes were positively regulated while 7 were negatively regulated in the presence of NifA. 11 out of 25 target genes were up-regulated under N_2_ fixation, as well, and 8 of them also possessed an upstream RpoN-binding motif. Correspondence to expression data is discussed below.

### Characterization of the NifLA Regulon: Transcriptional Activator for Nitrogen-fixation Related Genes

The transcriptional activator for *nifHDK* genes in *Azoarcus* sp. strain BH72 is the NifA protein, whose activity is known to be modulated by the anti-activator NifL in response to oxygen and combined nitrogen under free-living conditions [18]: the *nifLA* mutant BHLAO is unable to grow on N_2_. The NifA regulon has been analyzed only in root nodule endosymbionts up to now, such as *Bradyrhizobium japonicum* [46], *Sinorhizobium meliloti* [47] and *Rhizobium etli* [48]. In their regulatory network leading to symbiotic nitrogen fixation, low oxygen concentration is a major cue affecting NifA activity.

In order to globally assess the NifA regulon of strain BH72, we compared the transcriptome of wild type cells with that of the *nifLA* mutant strain BHLAO, both grown microaerobically under conditions allowing nitrogen fixation in presence of glutamate. The generation time of both strains was similar (wild type 2.7 h, BHLAO 2.6 h). The *nifL*::Ω strain (BHLAO) was previously constructed by marker-exchange mutagenesis, carrying a polar mutation in the *nifL* gene by insertion of a Sp/Sm-cartridge which also abolished *nifA* transcription [18]. BHLAO was not able to grow on N_2_ in the absence of combined nitrogen under microaerobic condition as NifA was essential for diazotrophy as transcriptional activator. It was also shown previously that *nifA* expression was undetectable in presence of combined nitrogen in strain BHLAO. To further verify that *nifA* expression is completely abolished in strain BHLAO under conditions of strong induction, total RNA was isolated from bacteria grown under N_2_ fixing condition in presence of 10 mM glutamate. As expected, a 0.421 kb amplification product of *nifA* was detected by RT-PCR only in wild type strain BH72 (Figure S2A) and not in strain BHLAO. Neither of the negative controls, without an RT step or template, respectively, generated amplification products. (Figure S2A). The use of equal amounts of RNA in both extracts was confirmed by semi-quantitative RT-PCR using primers for amplification of 16S rRNA (Figure S2B).

**Figure 4:**
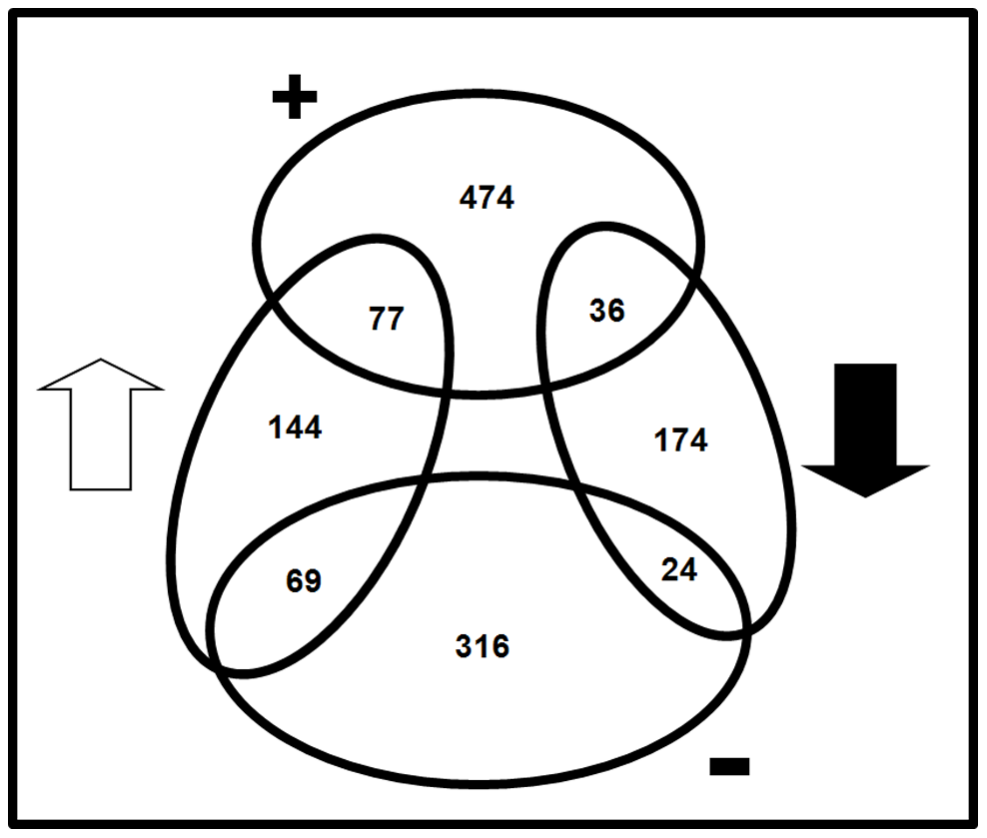
Venn diagram summarizing the number of differentially expressed genes of *Azoarcus* sp. BH72 from two different transcriptome studies (N_2_ response and NifA regulon) and their overlap. The numbers within the ovals represent genes modulated under the microarray experiments. Non-filled arrow and solid arrow represent up-regulation and down-regulation of genes under N_2_ fixation, respectively. The+or – symbols represent genes positively or negatively regulated by NifA, directly or indirectly, under N_2_-fixing condition. The circles and region of overlap are not drawn to scale.

The number of genes modulated in expression exceeded the number of genes modulated under N_2_ fixation. 996 genes differed in expression in the *nifLA* mutant in comparison to the wild type; 587 genes were positively regulated while 409 genes were negatively regulated (Table S1, Figure 4). The respective COG categories revealed that the putative regulon encompassed a broad range of functional categories; nearly all functional categories housed a fair number of candidates (Figure S3). This might partially be attributed to different growth conditions: these experiments had to be carried out with glutamate as a nitrogen source that does not repress *nif* genes, in contrast to the experiments comparing N_2_-fixing and ammonium-grown wild type cells without glutamate addition. Therefore, we analyzed the effect of addition of glutamate on the transcriptome of wild type BH72 under conditions of nitrogen fixation. The glutamate response affected only transcription of 6% (Table S1) of the genome, mostly at moderately high levels, compared to the BNF response (13%) or the NifA response (24%). Moreover, the genes modulated under the NifA response independent of nitrogen response were mostly independent of the glutamate response (see below). Therefore, metabolic effects of glutamate might not fully explain the strong impact of absence of NifA. This indicated a more global nature of NifA regulation beyond *nif-*gene regulation. The NifA regulon in rhizobia was also found to be relatively broad, with 323 genes differentially regulated under microaerobic, free living conditions in *B. japonicum* [46], or even 601 genes in symbiotic *S. meliloti* cells [47].

**Figure 5:**
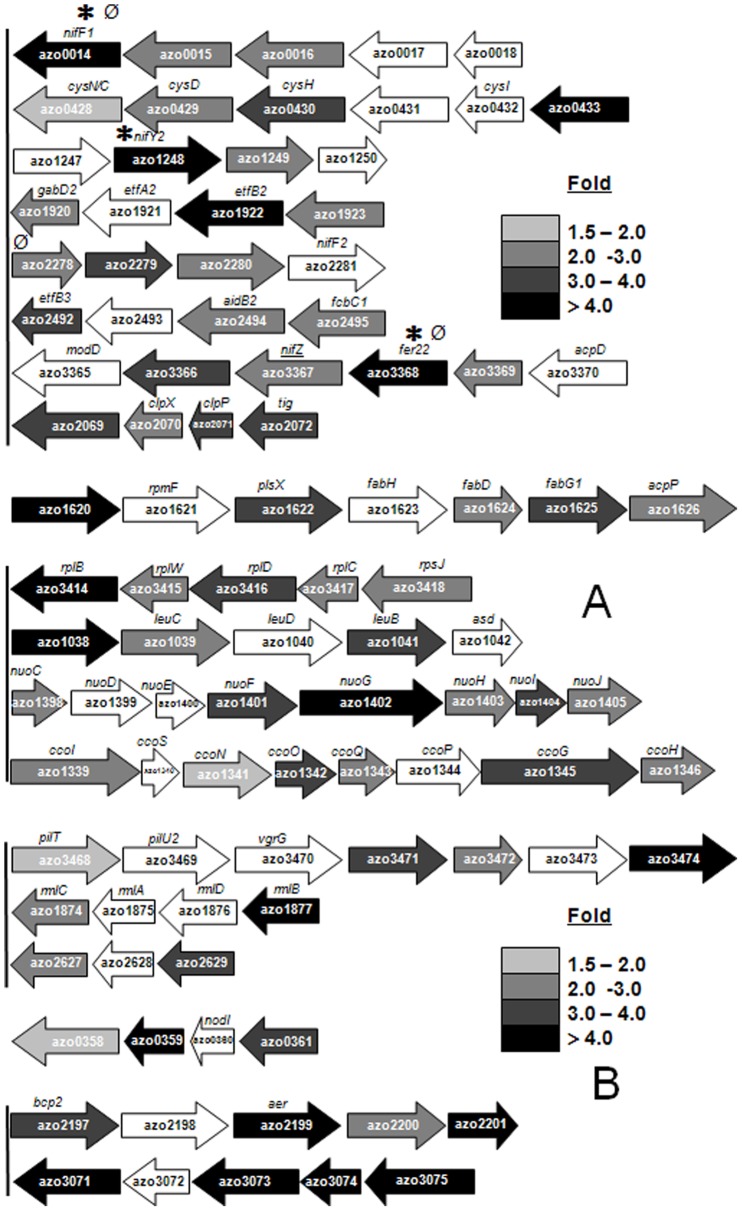
Gene clusters positively or negatively regulated in the presence of NifA under nitrogen fixing conditions. (A) Gene cluster(s) positively regulated by NifA and also up-regulated (upper), down-regulated (middle) or not modulated (lower) under N_2_ fixation. (B) Gene cluster(s) negatively regulated by NifA and also up-regulated (upper), down-regulated (middle) or not modulated (lower) under N_2_ fixation. Fold of induction of each gene is according to the gray shade code index provided. Blank arrows represent no detectable modulation of the target gene. Symbols ∗ or Ø represent predicted intergenic RpoN-binding sites or NifA-binding sites, respectively, upstream of the target gene of interest, as described in the legend of Figure 2.

As expected for NifA as the essential transcription activator for *nif* genes, the expression of *nif* structural genes as well as several of those involved in nitrogenase maturation were up-regulated under N_2_ fixation and severely repressed in the *nifA* mutant (Figure 2). Also *nif* related genes outside the *nif* cluster like *azo3366* and *azo3367* (*nifZ*) were found to be positively regulated by NifA (Table S1). Accordingly genes for electron transport complexes like Rnf1 (*azo0512* to *azo0517*) or genes encoding for mobile electron carriers like flavodoxin *nifF1* (*azo0014*) and ferredoxins like *fer22* (*azo3368*) were positively regulated by NifA under conditions of nitrogen fixation (Figure 5A, upper panel). Concordantly, RpoN and NifA binding sites were predicted in the upstream promoter region of *azo3368* and *nifF1,* respectively. Data from microarray experiments were corroborated with expression data obtained from real time PCR analysis: qRT-PCR analysis showed that indeed *azo3368* was strongly induced (90.7±15.4 fold) under N_2_ response and positively regulated (19.9±8.6 fold) by NifA (Table 1). As has been shown for *R. leguminosarum* [49], *hoxB* (*azo3807*) encoding for hydrogenase was also under NifA control in strain BH72. Proteases encoded by *clpX* (*azo2070*) and *clpP* (*azo2071*) up-regulated under N_2_-fixation were also found to be under NifA control, as in *Azotobacter vinelandii* [25]. As all genes of the operon were positively regulated by NifA and not affected by glutamate, this was not likely a metabolic effect.

Only 2 out of 10 transcriptional regulator genes, *gcv* (*azo3618*) and *azo0625*, up-regulated under N_2_-fixing conditions, were indeed positively regulated by NifA, albeit indirectly as no binding site was predicted (Figure S4, Table S1). No RpoN-binding site could be detected upstream of all these 10 genes up-regulated under N_2_ fixation (Table S1), thus modulation might be an indirect effect.

### The NifA Regulon Beyond Nitrogen Fixation

Despite the known role of NifA as transcriptional activator of N_2_-fixation-related genes, almost half of the modulated transcripts were under negative control in strain BH72 (409 versus 587 positively regulated); however, this was mostly indirect, since for the majority, NifA binding sites could not be predicted. Similar ratios were found in *B. japonicum* (190/133 genes) [46] or even in a reversed ratio in *S. meliloti* (291/320 genes) [47]. Moreover, in *Azoarcus* sp. strain BH72 only 77 or 24 of the genes up- or down-regulated under BNF, respectively, were under apparent positive or negative control of NifLA (Figure 4, Table S1). For example, as in *B. japonicum* [50], the fatty acid biosynthesis cluster (*azo1620– azo1627*) in strain BH72 was strongly down-regulated under N_2_ fixation but still, probably indirectly, activated by NifA (Figure 5A, middle panel). In accordance to expression data from microarray analysis, qRT-PCR further validated the *azo1625* modulation, with down-regulation under N_2_ response by -3.8±1.4 fold and activation by NifA by 52.4±16.6 fold.

Although the expression of the gene cluster encoding for a T6SS was enhanced under N_2_ fixation, it appeared to be independent of NifA control. In contrast, gene expression of 4 out of 22 secretory proteins, *azo0483*, *azo1306*, *azo2346* and *azo3872*, which were up-regulated under N_2_ fixation, were positively affected by NifA, probably indirectly (Table S1).

The majority of the NifLA-modulated genes (474 under positive, 316 under negative control) were not significantly affected by the nitrogen stimulus (Figure 4). This was unexpected, as NifA activity in NifL-containing *Gammaproteobactera* [51] and strain BH72 [18] is known to be controlled by the anti-activator NifL which inhibits the transcriptional activity of NifA by direct stochiometric interaction in response to elevated levels of fixed nitrogen and oxygen. Thus, one would expect regulation of genes in response to nitrogen and under control of NifA to be widely overlapping. Apparently, NifA plays an additional role under microaerobic conditions independent from this regulatory circuit. NifA in strain BH72 was shown to be functionally inactive in *nifDHK* activation in presence of combined nitrogen due to the binding of anti-activator NifL, and to be only active under N_2_ fixation [18]; however, it had been observed that *nifH* expression was severely repressed albeit not completely abolished under N surplus [6], [18], indicating slight residual activity of NifA. The availability of few free NifA (not bound to NifL) molecules might be sufficient to affect expression of downstream target genes. Moreover, *nifA* expression is detected even in the presence of combined nitrogen in strain BH72 and enhanced only 3 fold under N_2_ fixation [18].

According to our set parameter for the fold change of induction with 1.8 or above, the NifA regulon encompassed 24.9% of the genome. Setting the fold change threshold to 3.5 to cover only strongly modulated genes, drastically reduced the number of modulated targets to 4.4%. Many of these differentially expressed genes that were not modulated by ammonia addition had these relatively high levels of modulation: 67 genes were up-regulated and 48 down-regulated, respectively. However, for none of these down-regulated promoters a NifA binding site was predicted (except for *azo0499*), which indicates an only indirect mechanism of regulation.

Among those 67 activated genes, 17 genes encoded for hypothetical proteins. Additionally, NifA affected the expression of *sodC* (*azo0522*) (superoxide dismutase) related to oxygen stress, and *surE (azo1087)* (exopolyphosphatase). In *R. etli*, a peroxidase expression is shown to be under RpoN and NifA control [52]. NifA binding sites could also be predicted upstream of both the genes. Some of the strongly induced gene clusters included genes for metabolic processes such as *azo1003-azo1005* (transhydrogenases), *azo1038-azo1043* (leucine/isoleucine biosynthesis), *azo3418-azo3397* (ribosomal protein subunits) (Figure 5A, lower panel), and were not affected in expression by glutamate. Although no obvious RpoN or NifA binding sites were predicted upstream of the gene clusters encoding for respiratory chain components, expression of *nuo* genes comprising the NADH:ubiquinone oxidoreductase gene cluster (complex I) as well as *cco* genes comprising the high affinity terminal oxidase (*cbb_3_*-type) gene cluster (complex IV) both were positively regulated in presence of NifA, respectively (Figure 5A).

Negative regulation by NifA on targets with enhanced expression under nitrogen fixation seems to be quite unusual. Interestingly 9 out of 18 target genes in this category belonged to hypothetical proteins, but were mainly indirectly regulated by NifA according to the binding site prediction. For example, *azo3471* encoding for a conserved hypothetical protein was 11–fold induced by N_2_ but about 3.1 -fold negatively regulated by NifA (Table S1). qRT-PCR analysis corroborated a similar tendency of induction of *azo3471*∶21.2±9.6 fold induction under N_2_ response and 3.2±1.9 fold negative regulation by NifA. Also here, no effect of glutamate was detected. Others from this category included genes involved in phospholipid biosynthesis (*cfa1*), PHB granule association (*azo3815*), chemotaxis (*parA3*), maintenance of cell wall integrity (*rmlB*) and tRNA binding (*smpB*). Enhanced expression of PHB associated phasin under N_2_ was also observed in the *P. stutzeri* transcriptome [21]. A hybrid sensor response regulator *(azo1584*) and alternative sigma factor *rpoN2* (*azo1790*) strongly expressed under this condition might also have some regulatory roles (Figure S4).

Only 2 genes were repressed under N_2_ fixation and negatively regulated by NifA (Table S1): a hypothetical membrane protein (*azo0359* Figure 5B, middle panel) and assimilatory nitrite reductase *nirB* (*azo1175*). An RpoN-independent, NifA mediated regulation of respiratory nitrate reductase *nirK* has also been reported for *B. japonicum* [53]. Out of the 48 genes which were negatively affected by NifA in a nitrogen independent manner (Table S1), 25 are hypothetical or conserved hypothetical proteins (Table S1; Figure 5B, lower panel). Genes involved in amino acid uptake as well as branched chain amino acid metabolism were found to be repressed in the wild type as compared to strain BHLAO, along with urease and isocitrate lyase (key enzyme for glyoxalate pathway), indicative of down-regulation of pathways for alternative nitrogen sources and gluconeogenesis. The expression of *nodD* (transcriptional regulator of lysR fam) was strongly up-regulated in *nifLA ^–^* background which can also indirectly regulate the transcription of target genes under such conditions (Table S1).

Beyond its role as essential transcription factor for *nif* genes, NifA was suggested to control other genes involved in nodule maturation and bacteroid persistence in symbiotic bacteria, as well [54]. A generalized role of NifA in the nodulation competitiveness of *R. meliloti* had also long been reported [55]. In *Klebsiella pneumoniae*, the *nifA* product can substitute for the *glnG* (*ntrC*) product (two component response regulator) as a nitrogen control regulator, replacing the *ntrC* product in the activation of its own promoter unidirectionally and, in addition, the promoters of several nitrogen assimilation genes, including the *hut, put, aut*, and *glnA* genes [56]. Even NifA can substitute NtrC binding of upstream activating sequences [56].

Considering the strongly modulated (≥ 3.5 fold) 31 genes which were positively regulated by NifA and even up-regulated under N_2_ fixation, 7 were predicted to bear NifA binding upstream elements (Table S3). These may represent the real targets of NifA, genes with *nif* associated function. Perhaps NifA has a direct action on these target genes, by upstream binding. Those with binding sites and negatively regulated by NifA were independent from the N_2_ response. One of them, the putative repressor *frlR*, might be directly regulated by NifA and thus contribute to indirect regulation by NifA.

Comparing the array of 38 target genes with upstream RpoN-binding site (all modulated in their expression under N_2_ fixation) with the list of 25 genes with predicted NifA binding site (expression regulated by NifA in all) generated an overlap of 8 genes, in which RpoN binding promoter sequence as well as NifA binding upstream activating sequence occurred simultaneously. As expected, 4 (*rnfA1, azo0536, hesB, and nifE*) out of these 8 target genes were located within the *nif* cluster. Two out of the remaining genes situated outside the *nif-*cluster, encoded for mobile electron carriers involved in N_2_ fixation: *nifF1* (*azo0014*) encoding for probable flavodoxin and *fer22* (*azo3368*) encoding for probable ferredoxin. The other 2 encoded for hypothetical protein (*azo3080*) and conserved hypothetical ATPase (*azo3791*), respectively.

### Conclusion

The N_2_ response of *Azoarcus* sp. strain BH72 resulted in up-regulation of 144 genes and down-regulation of 174 genes, independent of the NifA regulon accounting to 61% of the response. Outside the *nif* related genes there is a significant up-regulation of genes for hypothetical proteins and non-*nif* related gene clusters and down regulation of several general metabolism-related genes under N_2_ fixation in strain BH72. The life style may undergo drastic changes when free-living bacteria become plant associated. Interestingly, the N_2_ response itself may partially act as a stimulus to trigger such changes, even in the free living state: In addition to the up-regulation of genes involved in the nitrogen fixation machinery, several protein secretion systems, motility appendages and hypothetical proteins likely to be colonization-related became up-regulated. On the other hand, genes particularly related to cell structure, energy metabolism and protein synthesis were found to be down-regulated under N_2_, perhaps correlating with the slow-down of general metabolism associated with nitrogen-fixing life style at slower growth. Furthermore, down-regulation of selected aromatic aminoacid (phenylacetic acid) degradation pathways under N_2_ stimulus observed in this study could be beneficial for successful establishment as disarmed pathogen in plant roots. This report provides for the first time evidence for a genome-wide regulatory activity of NifA in an endophyte. Although N_2_ fixation modulated 13% of the genome, the NifA regulon was found to encompass a broader and more diverse range of targets than ever expected, accounting to 24% of the genome or still 4.4% when only the strongly modulated (≥ 3.5 fold) genes were considered. Considering the large number of differentially expressed genes and the relatively few genes that may underlay direct regulation by NifA, it is tempting to speculate that the regulatory role of NifA is extended by the control of certain transcriptional factors such as sigma factors, activators or repressors. Several good candidates for further investigations are a*zo0625*, *gcvA*, and sigma factors like *algU* and *rpoN2* which are all modulated under N_2_ response and apparently indirectly transcriptionally regulated by NifA.

## Materials and Methods

### Strains and Growth Conditions

*Azoarcus* sp. strain BH72 was grown in an oxygen-controlled bioreactor (Biostat B; B. Braun Melsungen AG, Melsungen, Germany) at 37°C with stirring at 600 r.p.m, under microaerobic conditions (0.6% oxygen concentration) either in nitrogen free SM-medium on N_2_ [57], or in the same medium supplemented with either 10 mM ammonium chloride as N-source (N-replete) or with 10 mM glutamate (poor nitrogen source). A *nifLA^−^* mutant of *Azoarcus* sp. strain BH72 was constructed by disrupting the ORF of *nifL* by insertion of a Sp/Sm resistance cartridge [18]. Cells were harvested at an OD_578_ of 0.6 by centrifuging 45 mL of culture per tube at 9000×g for 5 min at 22°C. Pellets were immediately frozen in liquid nitrogen and stored at −80°C.

### *Azoarcus* sp. Strain BH72 Transcript Profiling Using a Genome-wide Oligonucleotide Microarray

Frozen bacterial cell pellets were re-suspended in a 1∶1 mixture of pre-warmed phenol-chloroform (pH 4.7) and 50 mM sodium acetate/10 mM EDTA/1% SDS (pH 5.1). Cells were incubated for 5 min at 65°C followed by 10 min of incubation on ice. For phase separation, the mixture was centrifuged for 10 min at 12°C and 8000×g, and the upper phase was mixed with the same volume of phenol-chloroform (pH 4.7). This phenol extraction was repeated three times followed by extraction with chloroform-isoamyl alcohol (24∶1). RNA precipitation was performed with the same volume of isopropanol for 45 min on ice. The RNA was pelleted by centrifugation (130000×g, 10 min, 4°C) and washed with 70% ethanol. RNA was dissolved in 1×RNAsecure (Applied Biosystems), snap frozen in liquid nitrogen and stored at −80°C until further processing.

Contaminating DNA was removed from RNA preparations by DNase I using Qiagen columns (RNeasy Mini Kit, Qiagen, Hilden, Germany) according to manufacturer’s instructions. The DNase I treated RNA was eluted twice in 30 µL of elution buffer, pooled and finally stored in 1×RNA secure in −80°C.

For transcriptome microarray analysis, 20 µg of total RNA was reverse transcribed with BioScript RT in respective reaction buffer (Bioline) with random hexamers and 4∶1 aminomodified-dUTP/dTTP nucleotide mix for 90 min at 42°C, after it had been controlled for DNA contamination by PCR. For coupling of fluorescent dyes to the aminoallyl-labelled first strand cDNA, aliquots of Cy3-NHS or Cy5-NHS esters (GE Healthcare) were dissolved in the elution of first strand cDNA, mixed and incubated for 90 min at room temperature in the dark. Blocking of all remaining dyes (quenching) was achieved by adding hydroxylamine (4 M) to the sample solution, followed by incubation for 15 min at room temperature. Subsequent cleaning up of labelled cDNA from remaining dyes was performed with the CyScribe GFX Purification Kit (GE Healthcare) according to manufacturer’s instructions. The Cy5- and Cy3-labelled cDNA that was used in one hybridization experiment were cleaned up together. The labelled cDNA was stored at −20°C until microarray hybridization after it had been checked for successful labeling [20]. The combined Cy3/Cy5-labelled targets were dried in a vacuum concentrator and further dissolved in DIG Easy hybridization solution (Roche, Mannheim, Germany) prior to hybridization. The transcriptome microarray spotted on epoxysilane-coated Nexterion Slide E (Schott) (CeBiTec, University Bielefeld) [20] contained 70mer oligonucleotide probes for the 3,989 predicted protein-coding genes from *Azoarcus* sp. strain BH72 in four replicates [20]. Before hybridization, the targets were denatured at 65°C for 10 min. The hybridization of fluorescently labelled cDNA targets to oligonucleotide microarrays was performed at 42°C for 14 to 18 hours as described previously [20]. The Cy3- and Cy5-fluorescence was scanned at 532 nm and 635 nm with the GenePix Scanner 4000A (Molecular Devices, Sunnyvale, CA, USA) with a pixel size of 10 µm. The image analysis was performed with the GenePix 4.1 program. After scanning, raw data images of the slides that contain information about gene expression levels were obtained by the GenePix software. These images were analyzed by identifying each spot on the array (with the help of an array layout template) with measurements of its fluorescence intensity and the corresponding background intensity. With the file transformation tool Express Converter v.2.1 (http://www.tm4.org/utilities.html), data files generated from GenePix (.gpr) were converted to TM4 files (.mev) that could be uploaded to the MIDAS platform. Normalization with MIDAS v2.19 (TM4 suite) was done in order to reduce variability by appropriately adjusting the data. In this study, the LOWESS (locally weighted scattered plot smoothing) normalization was performed using the block mode as set-up parameter as this mode allowed correction of systematic spatial variation between the grids of the array. The normalized data for each spot were exported to Excel for further downstream analysis. Overall, three independent experiments (biological replicates) were performed. Average expression fold were determined from normalized values of the three sets. Statistical analysis was performed by one-tailed paired t-test with Bonferroni correction for three independent experiments including dye-swap. Only genes that showed an expression of at least 1.8 fold and a P-value ≤0.05 were regarded as being differentially expressed. The microarray data have been deposited in the GEO database (http://www.ncbi.nlm.nih.gov/geo) under accession no. GSE49394.

### RT-PCR and Semi Quantitative RT-PCR

For two step RT-PCR, initially 200 ng or 10 ng DNaseI treated total RNA was reverse transcribed at 50°C for 1 hour using gene specific reverse primers: *nifA* with nifArev3RT (5′TCGTCCAGGTGCTCGCGGCTG 3′) or 16S rDNA with R518 (5′ATTACCGCGGCTGCTGG 3′), respectively, using SuperScript® III Reverse Transcriptase (RT) (life technologies) according to manufacturer’s instruction. Subsequent PCR amplification of the *nifA* cDNA in the second step was carried out with DreamTaq DNA polymerase (Thermo SCIENTIFIC) using an aliquot from the previous reverse transcriptase reaction as template and primers nifAfor1RT (5′ATGAGCGCGGCCGGTCCGATG 3′) and nifArev2RT (5′CACGGTTTCGTGCCCGGCGCG 3′) for 35 cycles of 1 min 95°C, 1 min 65°C, 1 min 72°C [18]. For 16S cDNA amplification, semi quantitative RT-PCR was carried out in a similar way using forward primer F341 (5′CCTACGGGAGGCAGCAG3′) and the previous reverse primer R518 for PCR amplification at 1 min 95°C, 1 min 50°C, 1 min 72°C [58] with samples being taken out at different cycle numbers (13–19) from the PCR reaction. In each case the products were separated on 1.5% agarose gel.

### Real-time PCR

For quantitative real-time RT-PCR of selected genes, synthesis of cDNA was achieved by using gene specific reverse primers for genes *azo1790*, *azo3471*, *azo0954*, *azo3368*, *azo0804*, *azo0805*, *azo1566*, *azo1625, azo720, azo0585, azo1077, azo3155, azo3246* and 16S rRNA by using the Verso 2-Step QRT-PCR Kit from ABgene. cDNA synthesis on 30 ng RNA and the quantitative PCR step with 1×2-Step QPCR Mix (ABgene) and 0.5×SYBR green I dye (Molecular Probes) was carried out as previously described [20]. The 2^−DD*C*^_T_ method was applied for data analyses, and 16S was used as a reference gene with the following primers: 16SRTfor: CTTGACATGCCTGGAACCTT, 16SRTrev: ATGACGT GTGAAGCCCTACC, azo1790for: AGCGGGTGTTCTTCAGCTAC, azo1790rev: GGCGCGAAAAGAAATACTTG, azo0954for: GTTTGTCAGTGCGCTTGGTA azo0954rev: GTCTTGTCGGCTTCGTTGAT, azo3471for: ATCGCCGACAACTATGTCCT, azo3471rev: CGCACGTTGAGGATGATTT, azo3368for: CCTTCCTCGAAGCCTTTCA, azo3368rev:TGTGCTCGTCGATGATGGTG, azo0804for: GTATCTCGCCCTCGGAAAC, azo0804rev: GCTGT, azo0805for: CGTCGAGGAGGTGGTGTATT, azo0805rev: CGCACGTTGAGGATGATTT, azo1566for: GAAGACCGAGCTGTGCAAGAC, azo1566rev: GACCTCGTCGAACAGGATGAC, azo1625for: GAAGTCGAG, azo1625rev: CGGAGGTGATGTTGATGATG; azo0720for: CTTCAAGCGTCGCAAGTTCT; azo0720rev: GTTGGTGCAGATCGGTGTAAG, azo0585for: CTCAAGAAGGCTTCCCACAG, azo0585rev: CAGGCAATCGGTGAAACC, azo1077for: ACGCATCACTCATTTCGTCA, azo1077rev: ACGCATCACTCATTTCGTCA; azo3155for: GCTCGTCGAGGTAGTCAAGG, azo3155rev: GTACGGTTTCGAGGATCAGC; azo3246for: ATCTGCTCCACGACCTGATT, azo3246rev: GATTGACGGGTACGAAGACG;

### Web Based Genome Wide Predictions

Genome wide prediction of RpoN binding sites was carried out by using the online tool PePPER (**P**rediction of **P**rokaryote **P**romoter **E**lements and **R**egulons): a web based Regulon, **T**ranscription **F**actor (TF) and **T**ranscription **F**actor **B**inding **S**ites (TFBS) mining system [59]. The regulon and TFBS database used by PePPER is based on DBTBS, RegulonDB and MolgenRegDB. TFBSs prediction from the PePPER tool box was used for the prediction of (RpoN-binding) considering all the listed known RpoN binding sites of the tool box in the intergenic regions of the annotated genome of *Azoarcus* sp. strain BH72 based on the MolGenRegDB of PWM models.

Genome wide NifA binding site prediction was carried out by using the net online programme Prodoric Database release 8.9 [60]. The new software suite Virtual Footprint version 3.0 was used for genome wide motif predictions. For prediction of NifA binding sites, Regulon Analyses mode was used. For each IUPAC code used, sensitivity threshold was set to 0.8 and core sensitivity was set at 0.9. The maximum distance to the downstream gene was set to 0.500 kb. The search was restricted to intergenic regions, and the option to remove redundant palindromic matches was also activated. The generated promoter sequences were considered only when the SEP-score was above -6.0. Intrinsic transcriptional terminators (azo_Terminator(s) were inferred from genome annotation of strain BH72 by GenDB [61].

## Supporting Information

Figure S1 **Sequence logo for genome wide RpoN-binding site of** ***Azoarcus*** **sp. strain BH72 created by using ‘WebLogo’.** The consensus motif is derived from individual motifs ranging from 46 to 49 bases predicted in the intergenic upstream region of target genes using the online webserver for prediction of prokaryotic promoter elements, PePPER. 173 putative RpoN-binding motifs upstream of 162 target genes with score >6.00 were used to create the logo with the WebLogo generator http://weblogo.berkeley.edu/.
(PDF)

Figure S2 **Analysis of** ***nifA*** **expression under N_2_ fixing condition in presence of glutamate.** (A) Analysis of *nifA* expression by RT-PCR. M, *Pst*I-digested λ DNA; A, wild type strain BH72; B, *nifL*::Ω insertion mutant BHLAO. In each case, reverse transcription reactions were carried out without (−RT; lanes 2 and 3), or with (+RT; lanes 4 and 5) Superscript III reverse transcriptase, and aliquots were used as templates for the subsequent PCR amplification, respectively. −T, no template control; +T, positive control with genomic DNA as template for PCR amplification. (B) Semi- quantitative RT-PCR analysis. As a quality control of the RNA, equal amounts (10 ng) of the same RNA preparations from samples A or B, respectively, were used for 16S rRNA-directed RT-PCR. 5 µl of the samples were taken after PCR amplification cycles 13 (lanes 4 and 5), 15 (lanes 6 and 7), 17 (lanes 8 and 9), or 19 (lanes 10 and 11), respectively, and the products were run on a 1.5% agarose gel. Negative controls (−RT): lanes 2 and 3.
(PDF)

Figure S3 **Distribution of differentially regulated genes of** ***Azoarcus*** **sp. strain BH72 according to COG categories.** Genes positively regulated by NifA under N_2_ fixing conditions shown by white bars and those negatively regulated by NifA under N_2_ fixing conditions are shown by black bars. (A) Broad COG categories; (B) subcategories within a category. C: Energy production and conversion, D: Cell cycle control, mitosis and meiosis, E: Amino acid transport and metabolism, F: Nucleotide transport and metabolism, G: Carbohydrate transport and metabolism, H: Coenzyme transport and metabolism, I: Lipid transport and metabolism, J:Translation, K: Transcription, L: Replication, recombination and repair, M: Cell wall/membrane biogenesis, N: Cell motility, O: Posttranslational modification, protein turnover, chaperones, P: Inorganic ion transport and metabolism, Q: Secondary metabolites biosynthesis, transport and catabolism, R: General function prediction only, S: Function unknown, T: Signal transduction mechanisms, U: Intracellular trafficking and secretion, no: not in COG.
(PDF)

Figure S4 **Transcription regulators up-regulated under N_2_ fixation in addition to** ***nifA*** **.** The width of the lines corresponds to relative lengths of amino acid sequence. The boxes on the lines represent respective pfam domains. The color shade of each line corresponds to the fold of induction under N_2_ fixation in microarray experiments. ∗, When present denotes that the expression of the target regulator is modulated by NifA, as well, in the microarray experiments. TMH: transmembrane helix.
(PDF)

Table S1 **Table listing all genes of** ***Azoarcus*** **sp. BH72 whose expression is modulated by 1.8 fold or more (P value < 0.05) in at least one of the three microarray experiments carried out in this work all conducted under microaerobic condition.**
(XLSX)

Table S2 **Predicted RpoN-binding sites for genes modulated according to Table S1 in** ***Azoarcus*** **sp. strain BH72.**
(XLSX)

Table S3 **Predicted NifA-binding sites for genes modulated according to Table S1 in** ***Azoarcus*** **sp. strain BH72.**
(XLSX)
