# Supplementary material for: Transcriptional Profiling of Nitrogen Fixation and the Role of NifA in the Diazotrophic Endophyte Azoarcus sp. Strain BH72
Source: PLoS One. 2014 Feb 6;9(2):e86527. doi: 10.1371/journal.pone.0086527 (PMC3916325; doi:10.1371/journal.pone.0086527)
Supplement: Figure S1 — Sequence logo for genome wide RpoN-binding site of Azoarcus sp. strain BH72 created by using ‘WebLogo’. The consensus motif is derived from individual motifs ranging from 46 to 49 bases predicted in the intergenic upstream region of target genes using the online webserver for prediction of prokaryotic promoter elements, PePPER. 173 putative RpoN-binding motifs upstream of 162 target genes with score >6.00 were used to create the logo with the WebLogo generator http://weblogo.berkeley.edu/. (PDF) [file pone.0086527.s001.pdf]

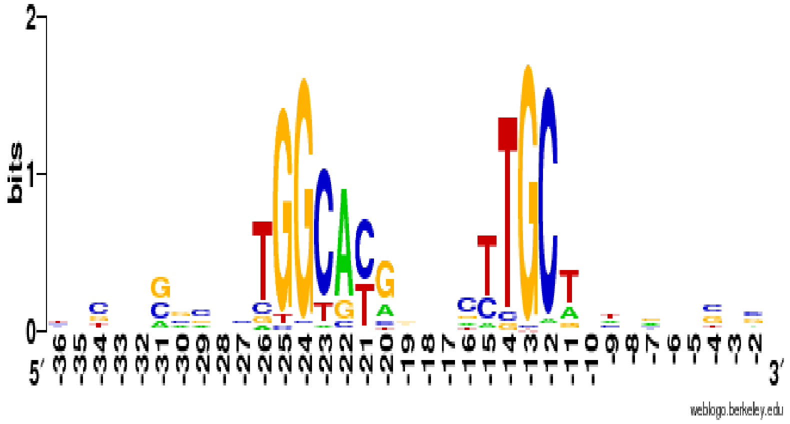

Figure S1. Sequence logo for genome wide RpoN-binding site of *Azoarcus* sp. strain BH72 created by using ‘WebLogo’. The consensus motif is derived from individual motifs ranging from 46 to 49 bases predicted in the intergenic upstream region of target genes using the online webserver for prediction of prokaryotic promoter elements, PePPER. 173 putative RpoN-binding motifs upstream of 162 target genes with score >6.00 were used to create the logo with the WebLogo generator http://weblogo.berkeley.edu/.
