# Supplementary material for: Transcriptional Profiling of Nitrogen Fixation and the Role of NifA in the Diazotrophic Endophyte Azoarcus sp. Strain BH72
Source: PLoS One. 2014 Feb 6;9(2):e86527. doi: 10.1371/journal.pone.0086527 (PMC3916325; doi:10.1371/journal.pone.0086527)
Supplement: Figure S2 — Analysis of nifA expression under N2 fixing condition in presence of glutamate. (A) Analysis of nifA expression by RT-PCR. M, PstI-digested λ DNA; A, wild type strain BH72; B, nifL::Ω insertion mutant BHLAO. In each case, reverse transcription reactions were carried out without (−RT; lanes 2 and 3), or with (+RT; lanes 4 and 5) Superscript III reverse transcriptase, and aliquots were used as templates for the subsequent PCR amplification, respectively. −T, no template control; +T, positive control with genomic DNA as template for PCR amplification. (B) Semi- quantitative RT-PCR analysis. As a quality control of the RNA, equal amounts (10 ng) of the same RNA preparations from samples A or B, respectively, were used for 16S rRNA-directed RT-PCR. 5 µl of the samples were taken after PCR amplification cycles 13 (lanes 4 and 5), 15 (lanes 6 and 7), 17 (lanes 8 and 9), or 19 (lanes 10 and 11), respectively, and the products were run on a 1.5% agarose gel. Negative controls (−RT): lanes 2 and 3. (PDF) [file pone.0086527.s002.pdf]

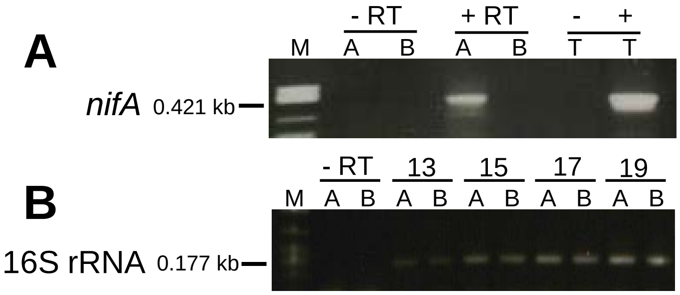

Figure S2. Analysis of *nifA* expression under N_2 fixing condition in presence of glutamate. (A) Analysis of *nifA* expression by RT-PCR. M, *Pst*I-digested λ DNA; A, wild type strain BH72; B, *nifL*::Ω insertion mutant BHLAO. In each case, reverse transcription reactions were carried out without (-RT; lanes 2 and 3), or with (+RT; lanes 4 and 5) Superscript III reverse transcriptase, and aliquots were used as templates for the subsequent PCR amplification, respectively. -T, no template control; +T, positive control with genomic DNA as template for PCR amplification. (B) Semi- quantitative RT-PCR analysis. As a quality control of the RNA, equal amounts (10 ng) of the same RNA preparations from samples A or B, respectively, were used for 16S rRNA-directed RT-PCR. 5 µl of the samples were taken after PCR amplification cycles 13 (lanes 4 and 5), 15 (lanes 6 and 7), 17 (lanes 8 and 9), or 19 (lanes 10 and 11), respectively, and the products were run on a 1.5% agarose gel. Negative controls (-RT): lanes 2 and 3.
