# Supplementary material for: Transcriptional Profiling of Nitrogen Fixation and the Role of NifA in the Diazotrophic Endophyte Azoarcus sp. Strain BH72
Source: PLoS One. 2014 Feb 6;9(2):e86527. doi: 10.1371/journal.pone.0086527 (PMC3916325; doi:10.1371/journal.pone.0086527)
Supplement: Figure S3 — Distribution of differentially regulated genes of Azoarcus sp. strain BH72 according to COG categories. Genes positively regulated by NifA under N2 fixing conditions shown by white bars and those negatively regulated by NifA under N2 fixing conditions are shown by black bars. (A) Broad COG categories; (B) subcategories within a category. C: Energy production and conversion, D: Cell cycle control, mitosis and meiosis, E: Amino acid transport and metabolism, F: Nucleotide transport and metabolism, G: Carbohydrate transport and metabolism, H: Coenzyme transport and metabolism, I: Lipid transport and metabolism, J:Translation, K: Transcription, L: Replication, recombination and repair, M: Cell wall/membrane biogenesis, N: Cell motility, O: Posttranslational modification, protein turnover, chaperones, P: Inorganic ion transport and metabolism, Q: Secondary metabolites biosynthesis, transport and catabolism, R: General function prediction only, S: Function unknown, T: Signal transduction mechanisms, U: Intracellular trafficking and secretion, no: not in COG. (PDF) [file pone.0086527.s003.pdf]

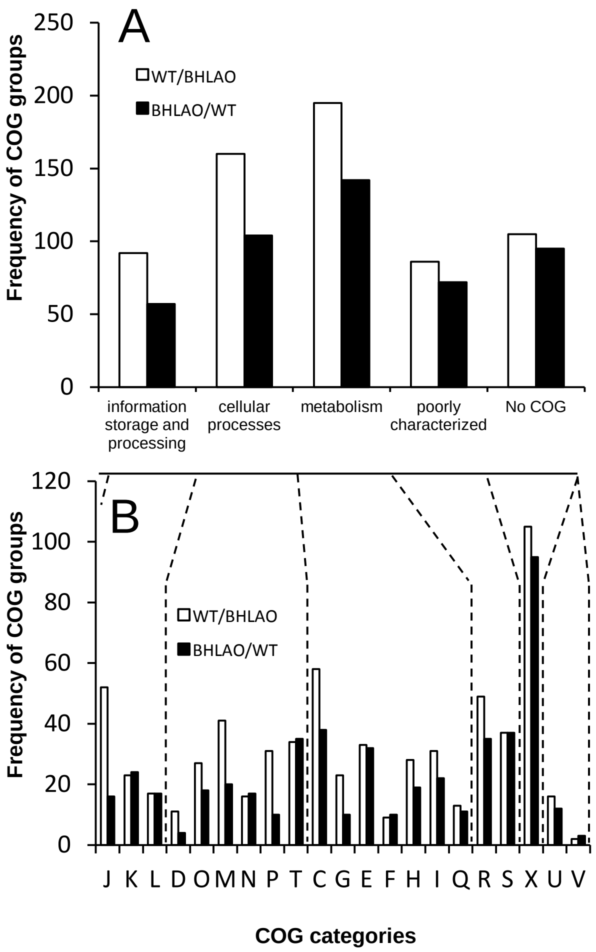

Figure S3. Distribution of differentially regulated genes of *Azoarcus* sp. strain BH72 according to COG categories. Genes positively regulated by NifA under N_2 fixing conditions shown by white bars and those negatively regulated by NifA under N_2 fixing conditions are shown by black bars. (A) Broad COG categories; (B) Sub categories within a category. C: Energy production and conversion, D: Cell cycle control, mitosis and meiosis, E: Amino acid transport and metabolism, F: Nucleotide transport and metabolism, G: Carbohydrate transport and metabolism, H: Coenzyme transport and metabolism, I: Lipid transport and metabolism, J:Translation, K: Transcription, L: Replication, recombination and repair, M: Cell wall/membrane biogenesis, N: Cell motility, O: Posttranslational modification, protein turnover, chaperones, P: Inorganic ion transport and metabolism, Q: Secondary metabolites biosynthesis, transport and catabolism, R: General function prediction only, S: Function unknown, T: Signal transduction mechanisms, U: Intracellular trafficking and secretion, no: not in COG.
