# Supplementary material for: Transcriptional Profiling of Nitrogen Fixation and the Role of NifA in the Diazotrophic Endophyte Azoarcus sp. Strain BH72
Source: PLoS One. 2014 Feb 6;9(2):e86527. doi: 10.1371/journal.pone.0086527 (PMC3916325; doi:10.1371/journal.pone.0086527)
Supplement: Figure S4 — Transcription regulators up-regulated under N2 fixation in addition to nifA . The width of the lines corresponds to relative lengths of amino acid sequence. The boxes on the lines represent respective pfam domains. The color shade of each line corresponds to the fold of induction under N2 fixation in microarray experiments. ∗, When present denotes that the expression of the target regulator is modulated by NifA, as well, in the microarray experiments. TMH: transmembrane helix. (PDF) [file pone.0086527.s004.pdf]

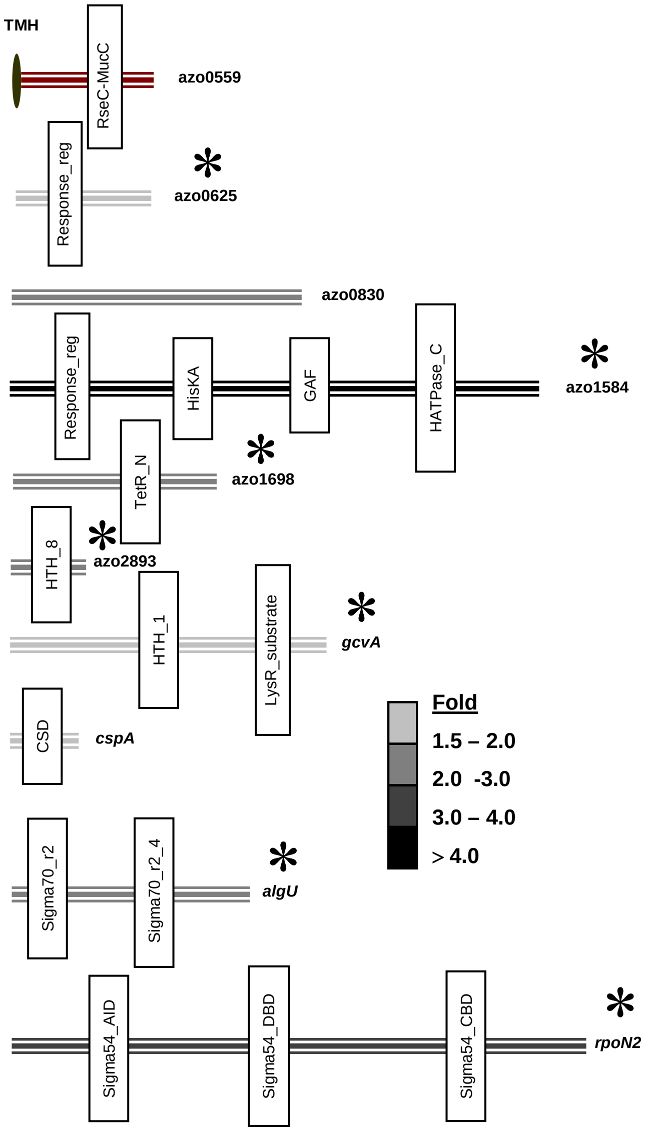

Figure S4. Transcription regulators up-regulated under N_2 fixation, in addition to *nifA*. The width of the lines corresponds to relative lengths of amino acid sequence. The boxes on the lines represent respective pfam domains. The color shade of each line corresponds to the fold of induction under N_2 fixation in microarray experiments. *, When present denotes that the expression of the target regulator is modulated by NifA, as well, in the microarray experiments. TMH: transmembrane helix
